# Genetic Diversity and Subspecific Races of Upland Cotton (*Gossypium hirsutum* L.)

**DOI:** 10.3390/genes15121533

**Published:** 2024-11-28

**Authors:** Asiya K. Safiullina, Dilrabo K. Ernazarova, Ozod S. Turaev, Feruza U. Rafieva, Ziraatkhan A. Ernazarova, Sevara K. Arslanova, Abdulqahhor Kh. Toshpulatov, Barno B. Oripova, Mukhlisa K. Kudratova, Kuvandik K. Khalikov, Abdulloh A. Iskandarov, Mukhammad T. Khidirov, John Z. Yu, Fakhriddin N. Kushanov

**Affiliations:** 1 Institute of Genetics and Plant Experimental Biology Academy of Sciences of Uzbekistan, Tashkent 111208, Uzbekistan; 2 Department of Genetics, National University of Uzbekistan, Tashkent 100174, Uzbekistan; 3 Research Institute of Plant Genetic Resources, National Center for Knowledge and Innovation in Agriculture, Tashkent 100180, Uzbekistan; 4 United States Department of Agriculture (USDA)—Agricultural Research Service (ARS), Southern Plains Agricultural Research Center, College Station, TX 77845, USA; john.yu@usda.gov

**Keywords:** upland cotton, *Gossypium hirsutum* L., subspecies and races, SSR markers, phylogenetic tree, intraspecific hybridization

## Abstract

**Background/Objectives**: The classification and phylogenetic relationships of *Gossypium hirsutum* L. landraces, despite their proximity to southern Mexico, remain unresolved. This study aimed to clarify these relationships using SSR markers and hybridization methods, focusing on subspecies and race differentiation within *G. hirsutum* L. **Methods**: Seventy polymorphic SSR markers (out of 177 tested) were used to analyze 141 alleles and calculate genetic distances among accessions. Phylogenetic relationships were determined using MEGA software (version 11.0.13) and visualized in a phylogenetic tree. ANOVA in NCSS 12 was used for statistical analysis. Over 1000 inter-race crosses were conducted to assess boll-setting rates. **Results**: Distinct phylogenetic patterns were identified between *G. hirsutum* subspecies and races, correlating with boll-setting rates. *Latifolium*, *richmondii*, and *morilli* showed no significant increase in boll-setting rates in reciprocal crosses. Cultivars Omad and Bakht, as paternal parents, yielded higher boll-setting rates. *Religiosum* and *yucatanense* displayed high boll- and seed-setting rates as maternal parents but low rates as paternal parents. Additionally, phylogenetic analysis revealed a close relationship between cultivars ‘Omad’ and ‘Bakht’ with *G. hirsutum* race *richmondii*, indicating their close evolutionary relationship. **Conclusions**: Reciprocal differentiation characteristics of *G. hirsutum* subspecies and races, particularly *religiosum* and *yucatanense*, should be considered during hybridization for genetic and breeding studies. Understanding the phylogenetic relationships among *G. hirsutum* taxa is crucial for exploring the genetic diversity of this economically important species.

## 1. Introduction

Cotton (*Gossypium* spp.), a member of the *Malvaceae* family, plays a vital role in the world economy as a source of natural fiber, food, and animal feed. Its origin, distribution, characteristics, and biological makeup have been the subject of extensive scientific investigation [1,2,3,4,5]. In their natural state, *Gossypium* species exist as perennial shrubs. However, concerted efforts by geneticists and breeders have successfully shortened their growing season, making them suitable for widespread annual cultivation [6].

Although significant scientific advances have been made in the taxonomy, evolution, and phylogenetic relationships within the *Gossypium* genus, particularly with the advent of genomic resources [7,8], debates on the precise relationships within the genus persist. Smith [9] proposed that the genus comprises forty-three species, with thirty-seven diploid (2n = 2x = 26) and six tetraploid (2n = 4x = 52) species. Other classifications, such as those by Fryxell [10], identified fifty species, while Brubaker et al. [11] and Percival et al. [12] suggested forty-nine species, including five tetraploid species. This discrepancy in the tetraploid species count stemmed from the discussion surrounding the classification of *G. lanceolatum* Todaro. Some proposed that it should be considered a race of *G. hirsutum* rather than a distinct species [13]. The *Gossypium* genus is recognized to encompass approximately fifty-threespecies, consisting of forty-six diploid species and seven allopolyploid species. Out of these diverse species, four have been domesticated for cultivation: two diploids, *G. herbaceum* (A_1_) and *G. arboreum* (A_2_), and two allopolyploids, *G. hirsutum* (AD_1_) and *G. barbadense* (AD_2_) [3,4,5,6,7,8,9,10,14,15,16,17,18].

Among the diverse tetraploid cotton species, *G. hirsutum* (AD_1_), also known as upland cotton, is distinguished by its widespread cultivation and genetic complexity [19]. It encompasses four recognized subspecies along with five independent forms whose precise taxonomic placement remains a mystery for researchers. The variations—race *latifolium*, race *richmondii*, race *morilli*, race *religiosum*, and race *yucatanense*—exhibit distinct characteristics that raise questions about their place within the *G. hirsutum* lineage.

The field of molecular biology provides scientists with an extraordinary array of approaches designed for analyzing genetic data [20,21,22,23]. These approaches empower scientists to delve into phylogenetic relationships, assess the extent of genetic diversity within populations, trace evolutionary lineages, and understand ecological adaptations [24]. The approaches and tools can unravel the complexity of genomic traits in plants and animals [20,21,22,23,24,25]. Within this molecular toolkit, PCR-based markers like Random Amplified Polymorphic DNA (RAPD), Amplified Fragment Length Polymorphism (AFLP), simple sequence repeat (SSR), and Single Nucleotide Polymorphism (SNP) have proven valuable [26,27,28,29]. Their ability to detect genetic variation within DNA sequences makes them ideal for investigating questions of genetic diversity and relationships between closely related taxa [30,31,32].

Despite advances in understanding *Gossypium* taxonomy, the phylogenetic relationships among the races and subspecies within *G. hirsutum*, particularly the placement of these independent forms, remain unresolved. This study addresses this gap by employing a combined approach of morphological characterization and SSR marker analysis to provide a more comprehensive understanding of the phylogenetic relationships within *G. hirsutum*. SSR markers also have proven to be a powerful tool for investigating genetic diversity and relationships in cotton [33,34]. This integrated approach, combining morphological and molecular data, will potentially lead to a refined classification of the independent forms within this species, contributing to a more accurate representation of its evolutionary history and informing future breeding efforts [35]. To achieve these objectives, this study utilizes the morphological characterization of the accessions and SSR markers.

## 2. Materials and Methods

### 2.1. Plant Materials

The seeds of all subspecies, races, and cultivars of the cotton species *G. hirsutum* L. studied within the framework of this research were sourced from the Uzbekistan Cotton Germplasm Collection, found at the Institute of Genetics and Plant Experimental Biology (IGPEB), Academy of Sciences of the Republic of Uzbekistan. The list of samples obtained from this collection is presented in Table 1.

### 2.2. Determining the Boll-Setting and Complete Seed-Setting Rates

To determine the phylogenetic relationships among *G. hirsutum* L. diversities, a series of intraspecific hybridization experiments were conducted. Knowledge of the fertility and complete seed-setting rates between these diversities is crucial for understanding their phylogenetic basis.

### 2.3. Genomic DNA Extraction

DNA extraction was performed using CTAB methods following Paterson et al. [36] with minor modifications. DNA quantity and quality were assessed using a NanoDrop 2000 Spectrophotometer system (Thermo Fisher Scientific, Waltham, MA, USA).

### 2.4. PCR-Based SSR Genotyping

A total of 177 simple sequence repeat (SSR) markers were selected from CottonGen database (https://www.cottongen.org/data/markers, accessed on 20 February 2022). PCR-based genotyping of these SSR markers was conducted as outlined in previous studies [31,37]. PCR experiments were performed using a T100 Thermal Cycler (BIO-RAD, Hercules, CA, USA). Each 10 µL reaction contained the following: 2.0 µL 5 × Screen Mix, 1.0 µL DNA, 1.0 µL Primers (F/R), and 6.0 µL DNAse/RNAese free water.

The PCR process, using the Hot-Start program, begins with an initial denaturation step at 94 °C for 2 min. This is followed by 35 cycles consisting of denaturation at 94 °C for 20 s, annealing at 55–60 °C for 30 s, and elongation at 72 °C for 30 s. Finally, the final extension is performed at 72 °C for 5 min.

PCR products were visualized by gel electrophoresis using a 1.5–2% agarose gel (CondaLab, Madrid, Spain) and the GelDoc Go Gel Imaging System (BIO-RAD, CA, USA).

### 2.5. Characterization of Polymorphic SSR Markers

Polymorphism information content (PIC) and heterozygosity (He) were calculated for each SSR marker according to PowerMarker V3.0. PIC provides an estimate of the discriminatory power of a marker, while He reflects the degree of genetic variation within the population.

PIC values were calculated for each polymorphic marker according to Botstein et al. [38] using the following formula:

$$PIC=1-\sum_{i=1}^{n}P_{i}^{2}-\sum_{i=1}^{n-1}\sum_{j=i+1}^{n}2P_{i}^{2}P_{j}^{2}$$

where *p_i_*—the frequency of the *ith* allele at a given locus and *n*—the total number of alleles.

He was calculated by the following formula according to Nei, M. (1973) [39]:

$$H\mathrm{e}=1-\sum_{i=1}^{n}P_{i}^{2}$$

where *p_i_*—the frequency of the *ith* allele at a given locus and *n*—the total number of alleles.

### 2.6. Statistical Analysis

One-way ANOVA [40] was used to analyze the statistical significance of differences in the average number of full seeds per boll among the different *G. hirsutum* accessions. This analysis tested for statistically significant differences between the mean number of full seeds across the groups. The results of the phylogenetic analysis were visualized using Mega 11 software [41].

## 3. Results

### 3.1. Morphological Descriptions of G. hirsutum Subspecies and Races

This section presents detailed morphological descriptions of the ten Upland cotton accessions used in this study. These descriptions provide a comprehensive understanding of the phenotypic variation within these accessions and will be complemented by the molecular analyses presented in later sections of this manuscript. Morphological characterization is crucial for identifying and classifying different subspecies and races within *G. hirsutum*. The following descriptions highlight key morphological features, including stem characteristics, leaf morphology, flower structure, boll size and weight, and fiber properties.

**Figure 1:**
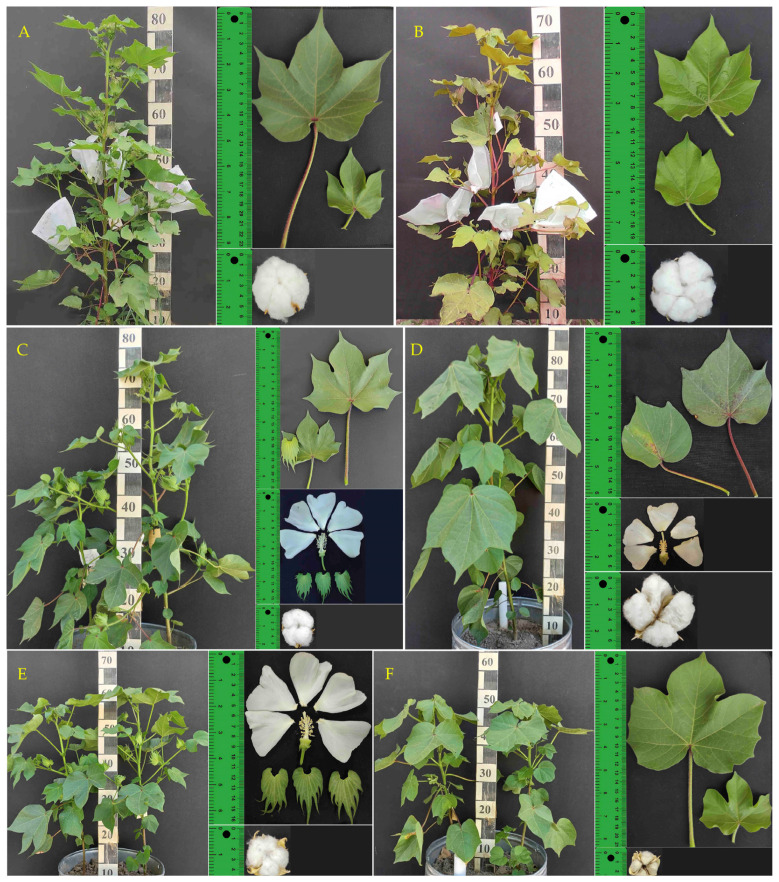
The studied subspecies and races of *G. hirsutum* L. (**A**) subsp. *euhirsutum* (Omad cultivar); (**B**) subsp. *euhirsutum* (Bakht cultivar); (**C**) subsp. *paniculatum*; (**D**) subsp. *mexicanum*; (**E**) subsp. *punctatum*; (**F**) race *yucatanense*.

Subsp. *euhirsutum*: The cultivar ‘Omad’ is characterized by larger cups and longer fibers. The main stem reaches a height of 95–110 cm, exhibiting medium hairiness and anthocyanin abundance (Figure 1A). Leaves are medium-sized (10.5 × 11.0 cm), claw-shaped with 3–5 lobes, and dark green in color. They are weakly hairy, with gossypol glands present on the underside, and have one nectar. Flowers have five yellow petals (5.1 × 4.4 cm). The calyx consists of three medium-sized, dark green, broadly heart-shaped petals with 11–14 teeth (6.3 × 3.2 cm), all covered with gossypol glands. The large cups contain 4–5 pieces; each boll weighs 6.5 g. The fiber is white and averages 31 mm in length. The cultivar ‘Bakht’ is described for its larger flowers, bolls, and seeds, growing on a 140–150 cm tall main stem with medium hairiness and anthocyanin abundance (Figure 1B). Leaves were medium-sized (11.5 × 12.0 cm), claw-shaped with 3–5 lobes, dark green, weakly hairy with gossypol glands on the underside, and had one nectar. The yellow petals were five in number (5.0 × 4.1 cm). The calyx had three medium-sized dark green broadly heart-shaped petals with 11–12 teeth (6.0 × 3.0 cm), all covered with gossypol glands. The large cups contained 4–5 pieces; each cotton boll weighed 6 g. The fiber was white and on average 30 mm in length.

Subsp. *paniculatum* (Figure 1C) is characterized as a tropical subspecies with medium to large flowers and more calyx teeth (11–13) compared to wild and ruderal types. The main stem is 100–110 cm tall, with medium hairiness and strong anthocyanin abundance. Leaves are medium-sized (10.5 cm), claw-shaped with 3–5 lobes, dark green, and medium hairy, with one nectar. Flowers have five yellow petals (5 × 3.5 cm). The calyx has three medium-sized, dark green, broadly heart-shaped petals with 11–13 teeth, all covered with gossypol glands. The medium-sized cups contain 3–4 pieces, and each boll weighs 3 g. The fiber is white and averages 22 mm in length.

Subsp. *mexicanum* is characterized by late maturation and a strong response to daylight, with a growth season of 205 days (Figure 1D). The main stem is 95–110 cm tall, exhibiting medium hairiness and strong anthocyanin abundance. Leaves are medium-sized (10 × 10.2 cm), claw-shaped with 3–5 lobes, dark green, and weakly hairy, with anthocyanin present. Each leaf has one nectar. Flowers have five yellow petals (5 × 3 cm). The calyx has three medium-sized, dark green, broadly heart-shaped petals with 9 teeth (2.5 × 1.7 cm), all covered with gossypol glands. The cups are small to medium-sized, containing 3–4 pieces, and the bolls weigh approximately 4 g. The fiber is white and averages 17 mm in length.

Subsp. *punctatum* (Figure 1E): As a ruderal subspecies, *punctatum* has average-sized flowers, bolls, and seeds. The main stem is 80–90 cm tall, with medium hairiness and strong anthocyanin abundance. Leaves are medium-sized (5 × 3.5 cm), claw-shaped with 3–5 lobes, dark green, and medium hairy, with weak anthocyanin presence. Each leaf has one nectar. Flowers have five yellow petals (4 × 3.5 cm). The calyx has three medium-sized, dark green, broadly heart-shaped petals with 8–9 teeth (3.5 × 1 cm), covered in gossypol glands. The medium-sized cups contain 3–4 pieces, and each boll weighs 4 g. The fiber is white and averages 20 mm in length.

Race *yucatanense* (Figure 1F) is distinguished by its small flowers, bolls, and seeds. The main stem is 95–100 cm tall, with medium hairiness and anthocyanin abundance. Leaves are medium-sized (9.5 × 10.5 cm), claw-shaped with 3–5 lobes, dark green, and weakly hairy, with one nectar. Flowers have five yellow petals (4 × 4 cm). The calyx has three medium-sized, dark green, broadly heart-shaped petals with 11–13 teeth (4.5 × 3 cm), all covered with gossypol glands. The small cups contain 3–4 pieces, and each boll weighs 3 g. The fibers exhibit a white phenotype, averaging 20 mm in length.

**Figure 2:**
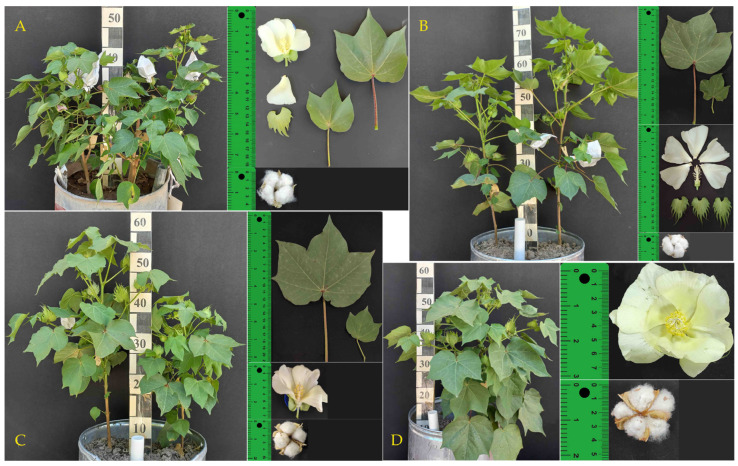
The studied subspecies and races of *G. hirsutum* L. (**A**) race *richmondii*; (**B**) race *religiosum*; (**C**) race *morilli*; (**D**) race *latifolium*.

Race *richmondii* is characterized by small flowers, bolls, and seeds (Figure 2A). The main stem is 95–100 cm tall, with medium hairiness and sparse anthocyanin abundance. Leaves are medium-sized (11.5 × 12.5 cm), claw-shaped with 3–5 lobes, dark green, and weakly hairy, with one nectar. Flowers have five yellow petals (3 × 2.5 cm). The calyx has three medium-sized, dark green, broadly heart-shaped petals with 11–13 teeth (3.5 × 2 cm), all covered with gossypol glands. The small cups contain 3–4 pieces; each boll weighs 3 g. The fiber is white and averages 23 mm in length.

Race *religiosum* (Figure 2B) is marked by its unique morphology, with medium-sized flowers, bolls, and seeds. The main stem is 80–90 cm tall, exhibiting medium hairiness and anthocyanin abundance. Leaves are medium-sized (10.0 × 10.5 cm), claw-shaped with 3–5 lobes, dark green, and weakly hairy, with one nectar. Flowers have five yellow petals (5 × 4 cm). The calyx has three medium-sized, dark green, broadly heart-shaped petals with 11–12 teeth (4.5 × 3.5 cm), all covered with gossypol glands. The medium-sized cups contain 3–4 pieces; each boll weighs 4.5 g. The fiber is white and averages 22 mm in length.

Race *morilli* is characterized by small flowers, bolls, and seeds. The main stem is 95–100 cm tall, with medium hairiness and sparse anthocyanin abundance (Figure 2C). Leaves are medium-sized (9.5 × 10.5 cm), claw-shaped with 3–5 lobes, dark green, and weakly hairy, with one nectar. Flowers have five yellow petals (3 × 3.5 cm). The calyx has three medium-sized, dark green, broadly heart-shaped petals with 11–13 teeth (4 × 2 cm), all covered with gossypol glands. The small cups contain 3–4 pieces; each boll weighs 3 g. The fiber is white and averages 23 mm in length.

Race *latifolium* (Figure 2D): With a main stem reaching 70–95 cm in height, *latifolium* exhibits strong hairiness and medium anthocyanin abundance. It features medium-sized flowers, bolls, and seeds. Leaves are also medium-sized (8 × 8.5 cm), claw-shaped with 3–5 lobes, dark green, and weakly hairy, with one nectar. Flowers have five yellow petals (4.5 × 4.5 cm). The calyx has three medium-sized, dark green, broadly heart-shaped petals with 6–8 teeth (3.2 × 3 cm), all covered with gossypol glands. The medium-sized cups contain 4–5 pieces; each boll weighs 3.5 g. The fiber is white and averages 23 mm in length.

Therefore, the morphobiological characteristics of ten different upland cotton accessions were studied, including two cultivars (‘Omad’ and ‘Bakht’) of subsp. *euhirsutum*, four other subspecies (*paniculatum*, *mexicanum*, *punctatum*, and *yucatanense*) and four races (*richmondii*, *religiosum*, *morilli*, and *latifolium*). These characteristics include stem height and hairiness, leaf size and shape, flower characteristics (petal number, size, and color), calyx features, boll size and weight, and fiber length. The descriptions highlight the diversity within *G. hirsutum*, noting morphology differences among the accessions.

### 3.2. Rates of the Boll-Setting and Seed-Setting in Cotton Hybrids

This study investigated the phylogenetic relationships within *G. hirsutum* by conducting over 1000 controlled crosses, encompassing both direct and reciprocal hybridizations across 25 distinct combination schemes. Successful hybrid progeny was generated from 37 combinations. Boll-setting rates (the proportion of pollinated flowers that developed into bolls) and complete seed-setting rates (the percentage of mature seeds formed within those bolls) were analyzed to assess cross-compatibility. This analysis encompassed diverse upland cotton accessions, including wild, ruderal, and cultivated types, with the latter being further categorized into tropical and temperate-adapted subspecies.

**Figure 3:**
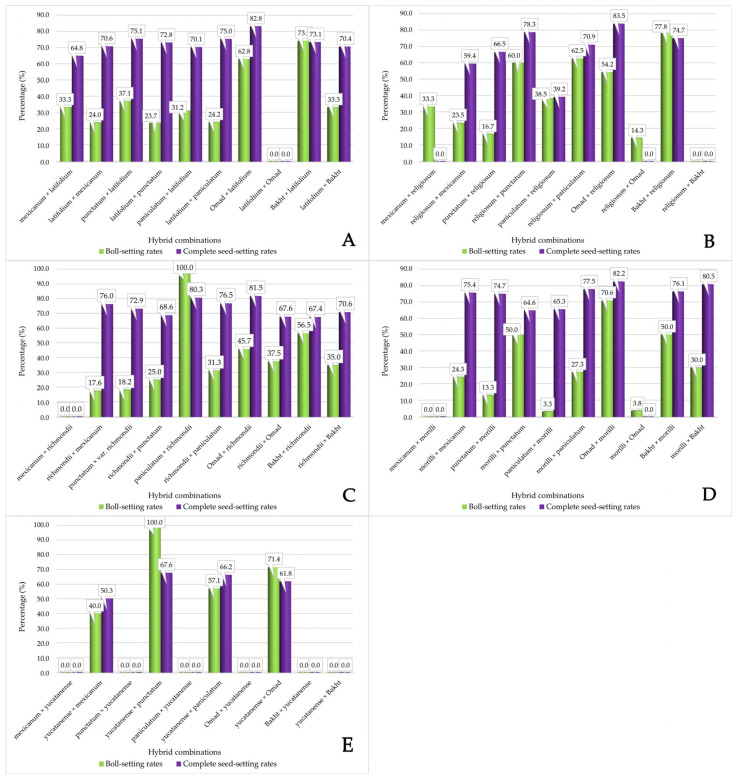
Boll- and seed-setting rates of the hybrids between *G. hirsutum* subspecies and races. (**A**) *G. hirsutum × latifolium*; (**B**) *G. hirsutum × religiosum*; (**C**) *G. hirsutum × richmondii*; (**D**) *G. hirsutum × morilli*; (**E**) *G. hirsutum × yucatanense*.

Hybridization experiments involving *G. hirsutum* subspecies and the race *latifolium* revealed significant variation in cross-compatibility, as evidenced by boll-setting rates (Figure 3A). The lowest compatibility was observed in crosses between *latifolium* and subsp. *punctatum* (23.7% boll-setting rate). Conversely, the highest compatibility within this group was noted between the cultivar ‘Bakht’ (subsp. *euhirsutum*) and *latifolium* (73.9% boll-setting rate). Other combinations, including *latifolium* × subsp. *mexicanum* and its reciprocal (24.0% and 33.3%, respectively) and *latifolium* × subsp. *paniculatum* and its reciprocal (24.2% and 31.2%, respectively), yielded similarly low boll-setting rates, indicating potential barriers to hybridization. The subsp. *euhirsutum* cultivar ‘Omad’ was characterized by a relatively high boll-setting rate (62.8%) when crossed with *latifolium*. However, when utilizing ‘Omad’ as the maternal genotype, the reciprocal crossing failed to produce any bolls. This observation suggests that cytoplasmic or maternal genetic factors could influence hybridization success. Complete seed-setting rates in successful hybrid bolls remained high across combinations, ranging from 64.6% to 82.2%, despite these variations in boll setting.

Experiments with hybridization between race *religiosum* and *G. hirsutum* subspecies revealed variable boll-setting rates (Figure 3B), with the lowest rates observed in race *religiosum* × ‘Omad’ (14.3%), subsp. *punctatum* × race *religiosum* (16.7%), and race *religiosum* × subsp. *mexicanum* (23.5%). The cultivar ‘Bakht’ × race *religiosum* crosses were remarkable in their boll-setting rates (77.8% success). Similarly, the race *religiosum* × subsp. *paniculatum* and race *religiosum* × subsp. *punctatum* crosses also showed promising rates (62.5% and 60.0%, respectively). Hybrid bolls from this group generally exhibited above-average complete seed-setting rates. The only exception was the subsp. *paniculatum* × race *religiosum* combination, where the complete seed-setting rate was lower (39.2%).

Hybridizing race *richmondii* with *G. hirsutum* subspecies generally resulted in boll-setting rates below 45.7% (Figure 3C). However, the two following combinations exhibited higher rates: subsp. *paniculatum* × race *richmondii* (100% boll-setting rate) and ‘Bakht’ × race *richmondii* (56.5% boll-setting rate). Despite the variable boll-setting rates, complete seed development within the resulting hybrid bolls remained consistently high across all combinations, ranging from 67.4% to 81.5%.

Cross-compatibility differences between the *G. hirsutum* subspecies and the race *morilli* were found to be reciprocal in hybridization studies, as assessed by boll-setting rates (Figure 3D). For example, *morilli* × subsp. *mexicanum* yielded a boll-setting rate of 24.3%, while the reciprocal cross, with subsp. *mexicanum*, as the maternal parent, failed to produce any bolls. Similarly, a pronounced reciprocal difference was observed in crosses with the ‘Omad’ cultivar. When ‘Omad’ served as the paternal parent, the boll-setting rate was 70.6%, whereas the reciprocal cross yielded a meager 3.8% boll-setting rate. This pattern of reciprocal differences, consistently observed across multiple *morilli* combinations, strongly suggests the influence of cytoplasmic or maternal genetic factors on hybridization success. Despite significant variability in boll-setting rates, successful hybrids from *morilli* crosses showed high fertility, with complete seed-setting rates between 64.6% and 82.2%. This suggests that, even with initial barriers to hybridization, viable and fertile progeny can be obtained from these inter-subspecific crosses.

In contrast to the *morilli* crosses, hybridization success was limited in crosses between the race *yucatanense* and *G. hirsutum* subspecies (Figure 3E). Viable hybrids were obtained from only four combinations: *yucatanense* × subsp. *mexicanum* (40.0% boll-setting rate), *yucatanense* × subsp. *paniculatum* (57.1% boll-setting rate), *yucatanense* × subsp. *punctatum* (100% boll-setting rate), and *yucatanense* × ‘Omad’ (71.4% boll-setting rate). Reciprocal crosses for these combinations and crosses between *yucatanense* and the cultivar ‘Bakht’ (both directions) failed to produce viable hybrids. This restricted hybridization success suggests strong reproductive isolation between *yucatanense* and certain *G. hirsutum* subspecies.

This research investigated the cross-compatibility of various *G. hirsutum* subspecies and races through controlled crosses. The study revealed significant variation in boll- and seed-setting rates, indicating the presence of reproductive barriers between certain groups. These barriers are influenced by factors such as flowering time, floral abundance, and cytoplasmic or maternal genetic effects. Despite these barriers, the high seed-setting rates in successful crosses suggest the potential for generating viable and fertile inter-subspecific hybrids. This information is valuable for understanding the genetic diversity and phylogenetic relationships within *G. hirsutum* and can be applied in cotton breeding programs.

### 3.3. Molecular and Phylogenetic Analysis of Gossypium Subspecies and Races

**Figure 4:**
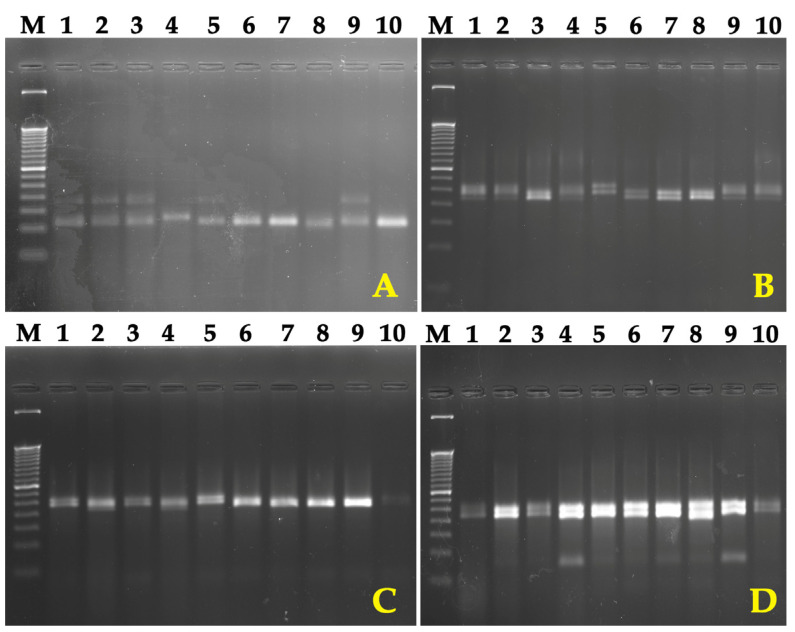
PCR-based analysis of cotton genetic polymorphisms using SSR markers. (**A**) Gh433; (**B**) NAU1221; (**C**) NAU1042; (**D**) NAU2265. M—molecular weight marker (base pairs, bp). (1) subsp. *mexicanum*; (2) subsp. *punctatum* (Fanome); (3) subsp. *paniculatum*; (4) race *latifolium*; (5) race *morilli*; (6) race *religiosum*; (7) race *yucatanense*; (8) race *richmondii*; (9) subsp. *euhirsutum* (cultivar ‘Omad’); (10) subsp. *euhirsutum* (cultivar ‘Bakht’).

This study utilized DNA marker technology, specifically simple sequence repeat (SSR) markers, to evaluate the genetic diversity of *G. hirsutum*. A PCR assay employing 177 SSR markers assessed DNA polymorphism among the cotton samples (Figure 4). Of the 177 primer pairs tested, 104 yielded amplified products, with 70 exhibiting polymorphism and amplifying a total of 141 alleles. The average number of alleles per SSR marker was 2.10. Three markers failed to amplify in any of the tested genotypes. These markers provided information on primer sequences, molecular weight range, polymorphism information content (PIC) values, heterozygosity (He) values, associated traits, chromosome numbers, and relevant references (Table S1).

PIC values for the polymorphic markers ranged from 0.18 to 0.48, indicating varying degrees of informativeness across loci. Heterozygosity (He) values ranged from 0.1638 to 0.500, reflecting the genetic diversity within the evaluated cotton genotypes. Several SSR markers were associated with fiber quality traits, including fiber strength (e.g., BNL2634, NAU2276), fiber elongation (e.g., TMB1268), fiber micronaire (e.g., Gh247), and fiber uniformity (e.g., CGR6078). Markers were also linked to stress-related traits, such as salt tolerance (e.g., CIR0246, BNL3594) and relative malondialdehyde content (e.g., BNL3424, NAU1151). Resistance to biotic stresses was associated with specific markers, including resistance to reniform nematode (*Rotylenchulus reniformis*) (e.g., TMB0426) and Verticillium wilt (*Verticillium dahliae*) (e.g., CIR0329). Associations were also found for other important agronomic traits such as seed cotton yield (e.g., JESPR204), plant height (e.g., NAU1028), and bolls per plant (e.g., BNL3594).

The SSR markers were mapped to specific chromosomes within the *G. hirsutum* genome, with contributions from both the At (A-subgenome) and Dt (D-subgenome). For example, fiber elongation was linked to markers on A06 and D05, while salt tolerance was associated with markers on D01. References for each marker are provided in Supplementary Table S2 to facilitate reproducibility and comparison with other studies. The markers used in this study have diverse origins, including those identified in previous cotton research.

Genetic distances between accessions were calculated using the polymorphic SSR markers. These data were then used to construct and visualize a phylogenetic tree. Phylogenetic analysis revealed two distinct clusters. The first cluster contained the *G. hirsutum* cultivars ‘Omad’ and ‘Bakht’, which displayed significant polymorphism and were closely related to the *G. hirsutum* race *richmondii*. This proximity suggests a closer relationship between these cultivars and *G. hirsutum* race *richmondii* than previously recognized.

**Figure 5:**
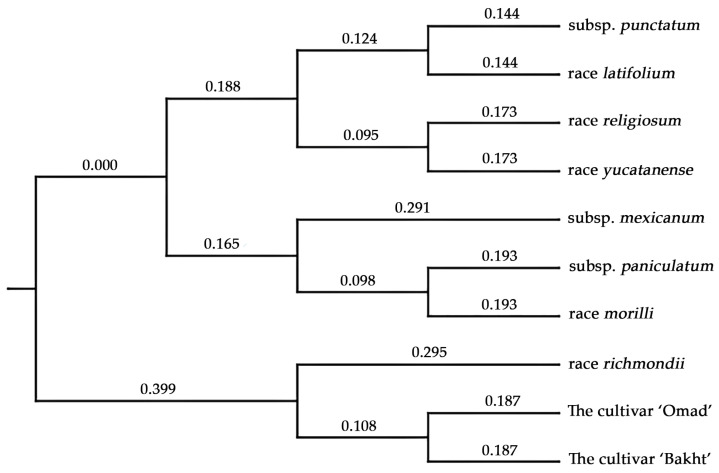
The phylogenetic tree of studied *G. hirsutum* accessions. Evolutionary relationships among subspecies and races of upland cotton are illustrated in this phylogenetic tree, which was constructed using the Neighbor-Joining method of the PAUP 4.0 (Phylogenetic Analysis Using Parsimony) program based on genetic distances calculated from 70 polymorphic SSR markers. The numerical values on the branches represent genetic distances, reflecting the degree of divergence between the accessions. A scale bar is included to indicate the proportional relationship between genetic distance values and the extent of evolutionary divergence.

The phylogenetic tree (Figure 5) illustrates the evolutionary relationships among various subspecies, races, and cultivars of *G. hirsutum*. Constructed as a rooted phylogram, branch lengths represent genetic or evolutionary distances.

The phylogeny demonstrates a clear separation of clades corresponding to wild, domesticated, and transitional taxa. The root represents the most recent common ancestor (MRCA) of all included taxa, with subsequent branching points reflecting divergence events within the evolutionary history of *G. hirsutum*.

Clade 1 comprises subsp. *punctatum*, race *latifolium*, race *religiosum*, and race *yucatanense*. These taxa exhibit close evolutionary relationships, evidenced by their relatively short branch lengths. This grouping suggests a shared evolutionary trajectory among these wild races, potentially influenced by ecological and geographic factors.

Clade 2 includes subsp. *mexicanum*, subsp. *paniculatum*, and race *morillii*. The intermediate branch lengths separating these taxa suggest moderate divergence, likely representing evolutionary transitions between wild and cultivated forms. *G. hirsutum* subsp. *mexicanum* occupies a basal position within this clade, indicating its divergence before the other members.

And clade 3 encompasses *G. hirsutum* race *richmondii* and the two cultivars (‘Omad’ and ‘Bakht’), classified under *G. hirsutum* subsp. *euhirsutum*. The relatively long branch leading to *G. hirsutum* race *richmondii* indicates greater divergence. In contrast, the minimal branch lengths between the ‘Omad’ and ‘Bakht’ cultivars suggest recent domestication and genetic similarity due to selective breeding.

The tree highlights the evolutionary continuum from wild forms to domesticated cultivars within *G. hirsutum*. The basal position of the domesticated clade suggests its divergence from ancestral wild races, with subsequent refinement under anthropogenic selection pressures. The close relationship between the ‘Omad’ and ‘Bakht’ cultivars indicates minimal genetic differentiation, supporting the hypothesis of a recent common ancestry or shared breeding origins.

Thus, understanding the phylogenetic relationships among *G. hirsutum* taxa provides critical insights into the genetic diversity of this economically important species. This information is invaluable for breeding programs to improve cotton yield, pest resistance, and environmental adaptability. Furthermore, delineating wild and domesticated lineages elucidates the cotton’s domestication history and evolutionary dynamics. This phylogeny underscores the genetic diversity and evolutionary relationships within *G. hirsutum*, offering a robust framework for future research on cotton improvement and conservation.

### 3.4. In Silico Annotation Results

To identify genes associated with important agronomic traits in *G. hirsutum* L., an in silico PCR analysis was conducted. This analysis focused on identifying putative genes located near SSR markers previously linked to fiber quality, salinity tolerance, and wilt resistance.

In silico PCR was performed using the sequences of 70 polymorphic SSR markers. Of these, 54 primer pairs successfully produced virtual PCR products (amplicons), while 16 did not. The chromosomal locations and amplicon sizes were determined for the successful primer pairs (Table S2). For example, primers Gh056, Gh064, and Gh110, which are associated with fiber quality, all produced amplicons of 124 bp in length. These primers are located in the D-region of the genome (D07, D12, D10). The remaining 16 markers did not produce amplicons, including Gh591 and CIR0329.

## 4. Discussion

Plant species often exhibit distinct morphological variations that can be used to differentiate taxa and investigate intraspecific diversity [42,43]. Hybridization patterns, in conjunction with morphological data, provide valuable insights into phylogenetic relationships and reproductive barriers [44]. This study analyzed hybridization rates among different *Gossypium* taxa to unravel the complex relationships within *G. hirsutum*.

Our findings revealed a general pattern of asymmetric hybridization success, with lower boll-setting rates observed when certain races, such as *latifolium* and *religiosum*, were used as the maternal parent. This parental influence on hybridization success mirrors observations in other tetraploid *Gossypium* species, such as *G. mustelinum* [37,44]. Similar patterns of asymmetric reproductive barriers have been documented in other plant genera, suggesting a potential role for factors such as cytoplasmic–nuclear interactions or maternal effects in reproductive isolation [45].

The observed variation in boll-setting rates in our study could be attributed to several factors, including asynchronous flowering among the accessions. Differences in flowering phenology and fecundity between cultivated and wild/ruderal *G. hirsutum* accessions further highlight the potential role of these traits in reproductive isolation and speciation. However, additional mechanisms, such as pollen incompatibility or stylar interactions, may also contribute to the observed patterns. Further research is needed to elucidate the precise mechanisms underlying these reproductive barriers and their implications for speciation within *Gossypium*.

The distinct phylogenetic placement of *G. hirsutum* race *religiosum* observed in our study, based on both hybridization patterns and molecular marker data, supports its classification as a separate species, *G. religiosum*, as proposed by Mauer [46,47,48]. This finding underscores the importance of integrating multiple lines of evidence, including reproductive compatibility, morphology, and molecular markers, to resolve taxonomic uncertainties in *Gossypium* [49,50]. Similarly, our results corroborate previous studies suggesting a close phylogenetic relationship between *G. hirsutum* race *morrilli* and *punctatum* [51] and highlight the complex evolutionary history of *G. hirsutum* race *yucatanense*.

## 5. Conclusions

This study provides a comprehensive analysis of the phylogenetic relationships within *G. hirsutum* by integrating morphological characterization and SSR marker data. Our findings reveal distinct clusters within this species, highlighting the complex evolutionary history of its subspecies and races. Notably, the broadleaf form of *G. hirsutum* and the ruderal subsp. *punctatum* form a single subcluster, while *G. hirsutum* races *religiosum* and *yucatanense* are identified as independent forms.

The observed high degree of cross-compatibility among certain *G. hirsutum* taxa has direct implications for breeding strategies aimed at enhancing hybrid vigor and expanding the genetic base of cultivated cotton. By identifying compatible crosses, such as those between cultivated varieties and race *richmondii*, breeders can effectively introduce desirable traits, such as disease resistance or improved fiber quality, from wild or underutilized races into elite cultivars. Furthermore, understanding the reproductive barriers, particularly those related to flowering time and pollination, can inform the development of targeted breeding strategies to overcome these barriers and facilitate gene flow between desirable genotypes.

This integrated approach, combining morphological and molecular data, provides a more robust framework for understanding the relationships within *G. hirsutum* and resolving taxonomic uncertainties. These findings not only contribute to our knowledge of cotton evolution but also have practical applications for cotton improvement. Future research should focus on further investigating the genetic basis of reproductive barriers, exploring the potential of underutilized *G. hirsutum* races for breeding, and applying genomic approaches to refine the phylogeny of this important crop species.

## Figures and Tables

**Table 1:** List of the *G. hirsutum* L. subspecies, races, and cultivars used in this study.

№	Accession Code	*G. hirsutum* Subspecies and Races	Country of Origin
1	AD1-(wild)-00299	subsp. *mexicanum*	Mexico
2	AD1-(wild)-00292	subsp. *punctatum* (Fanome)	Central and South America
3	AD1-(wild)-00313	subsp. *paniculatum*	Rhodesia
4	-	subsp. *euhirsutum* (Omad cultivar)	Uzbekistan
5	-	subsp. *euhirsutum* (Bakht cultivar)	Uzbekistan
6	AD1-(wild)-00303	race *latifolium*	Mexico
7	AD1-(wild)-00306	race *religiosum*	Mexico
8	AD1-(wild)-00300	race *richmondii*	Mexico
9	AD1-(wild)-00317	race *morilli*	USA
10	AD1-(wild)-00273	race *yucatanense*	Mexico

## Data Availability

Data sharing is not applicable to this article as no new data were created or analyzed in this study.

## Supplementary Materials

The following supporting information can be downloaded at www.mdpi.com/article/10.3390/genes15121533/s1, Table S1: The Polymorphic SSR markers, and their polymorphism information content (PIC) and heterozygosity (He) values. Table S2: Chromosomal locations and amplicon sizes of identified primers associated with fiber quality traits. References [52,53,54,55,56,57,58,59,60,61,62,63,64,65,66,67,68,69,70,71,72,73,74,75,76,77,78,79,80,81,82,83,84,85,86] are cited in the supplementary materials (Table S1).
