# Supplementary material for: Genetic Diversity and Subspecific Races of Upland Cotton (Gossypium hirsutum L.)
Source: Genes (Basel). 2024 Nov 28;15(12):1533. doi: 10.3390/genes15121533 (PMC11675639; doi:10.3390/genes15121533)
Supplement: Supplementary file 1 [file genes-15-01533-s001.zip › genes-3293947-Table S1.pdf]

**Table S1** Polymorphic SSR markers, and their polymorphism information content (PIC) and heterozygosity (He) values

| Marker | Forward primer (5'-3') | Reverse primer (5'-3') | Molecular weight (bp) | PIC | He | Associated trait | Chr. No* | Reference |
|---|---|---|---|---|---|---|---|---|
| Gh034 | CCCTTTTGTTATCTAACTTCTGTTA CTCCTAAC | CCTTTTGTTTAGCTCTTCTATACTTGA ATTCC | 135, 145, 150 | 0,331 | 0,420 | - | A13 | [51] |
| Gh056 | TCCATTAGACAAAGTTTTCTAAAGTTC | TGAGACTTCCAACCAGATACAG | 80 | 0,375 | 0,500 | Fiber quality | D07 | [47] |
| Gh064 | GAGAAGCCAATCCCATTTAAGA | GATAGTGCATCTCTAGAGAAGGA CA | 110, 130 | 0,375 | 0,500 | Fiber quality | A12 | [52] |
| Gh110 | ACCATCCCAAAGAATCATCCTC | ACTAAAACCAAGGCAATAAAGTG | 180, 190 | 0,364 | 0,480 | Fiber quality | D10 | [53] |
| Gh132 | TCATGGAACACCAAAGTTGGA | ACATGATAGATTATTCAGCAATGCA | 140, 145, 150, 155, 170 | 0,331 | 0,420 | resistance to reniform nematode | D11 | [54] |
| Gh247 | CTTCTTCCGCCACGTAAGTCC | CAGCCTAACCAAGAACCCAATCG | 130, 135, 145 | 0,372 | 0,495 | Fiber micronaire | A09 | [55] |
| Gh277 | TACTAAAACCAAGGCAATAAAGTGA | CACCACCTTCCATATATCTTGCTC | 100, 110, 115 | 0,293 | 0,357 | SF (short fiber) | D10 | [56] |
| Gh388 | CATCATCATCGTCGTCGCCG | GCAATGGAAGCTTTCGTCTCTTTC | 150, 415, 615, 930, 1210 | 0,374 | 0,499 | Fiber quality | A05 | [57] |
| Gh433 | TACCACATTGGATGTTTGCAAACCC | ATAGCAAACTGGAATCACTCCAAGC | 115, 165, 170, 180 | 0,351 | 0,455 | VW resistance | A06 | [58] |
| Gh591 | GATTTGAAAACTGGAGGCATCTCC | TCGGTTACCACCAATTTAACCAGC | 130, 145 | 0,351 | 0,455 | Fiber strength (FS) | A06 | [59] |
| DPL0050 | TTACTGAATCCTAGGCGAGATTGT | TTCTAGCCTCCACCTCGCT | 140, 145, 150 | 0,356 | 0,464 | VW resistance | D11 | [60] |
| DPL0131 | ACATACGGGTTGAAATGTACTCCT | ATGAATGCAGATCATTACGCCT | 180, 240 | 0,372 | 0,495 | Fiber elongation, fiber strength, | D11 | [61] |
| CIR0139 | AAACAAATGGAGAGGGT | ACCTGTGGTCTGCAAT | 160 | 0,375 | 0,500 | Fiber quality | D05 | [62] |
| CIR0246 | TTAGGGTTTAGTTGAATGG | ATGAACACACGCACG | 150, 155 | 0,351 | 0,455 | Salt tolerance | D02 | [63] |
| CIR0329 | AATATGTGAATGGCCCC | GCGGATTCTGCTGCT | 250 | 0,375 | 0,500 | VW resistance | A06/A02 | [60] |
| BNL0226 | TTATTCTCACAGCCGGAACC | TTCACCCTCTCGCTTCTCAT | 240, 250 | 0,18 | 0,1638 | FOV resistance | A03 | [64] |
| BNL1421 | TGAAGATTTGGAGGCAATTG | GAAATCAAGCCTCAATTCGG | 190, 210 | 0,351 | 0,455 | Lint cotton yield, micronaire | A13 | [65] |
| BNL1666 | TGTCAGAAAAGTTTTTCCAAGG | AGATCATATTTAAAAGAAAAAGAAAACC | 100, 120, 145 | 0,370 | 0,491 | Plant height, dry stem weight | A01 | [66] |
| BNL1694 | CGTTTGTTTTCGTGTAACAGG | TGGTGGATTCACATCCAAAG | 100, 110, 135, 150 | 0,319 | 0,398 | Seed index, fiber length | D16/A07 | [65,67] |

| | | | | | | | | | |
|---|---|---|---|---|---|---|---|---|---|
| BNL2634 | AACAACATTGAAAGTCGGGG | CCCAГCTГCTTATTГГTTTC | 240, 250, 270, 290 | 0,358 | 0,468 | Fiber length, fiber strength | D07 | [68] |
| BNL3140 | CACCATTGTGGCAACTGAGT | GGAAAAGGGAAAGCCATTGT | 160, 170 | 0,268 | 0,320 | Fiber strength | D23/A09 | [68] |
| BNL3424 | TGTGCCGTCTCAAAATGAAG | AAGACCAATCTGTTGCCAGC | 160, 180 | 0,364 | 0,48 | Relative malondialdehyde | D03 | [69] |
| BNL3436 | AACATAGCCTACCATTGCCG | TTGTTTGCCAAATTTGAAGC | 130, 180 | 0,372 | 0,495 | Fiber length, fiber micronaire | D25 | [68] |
| BNL3452 | TGTAACTGAGCAGCCGTACG | GCCAAAGCAGAGTGAGATCC | 190 | 0,375 | 0,5 | Relative malondialdehyde | D19/A05 | [69] |
| BNL3594 | AGGGATTTTGATTGTTGTGC | TGAATTCAAAACAAATGTTAGCC | 160, 170, 210 | 0,370 | 0,491 | Salt tolerance, salt_RMDA, SY (seed cotton yield), BN (bolls per plant) | A06 | [69,70] |
| BNL3601 | TTCCGTTGATGGAAATTGAA | ACAAGAATGCGTGTGTCTGC | 150, 165 | 0,351 | 0,455 | Maturity, cell wall thickness | A05 | [65] |
| BNL3792 | TTCGAGATCCCCTGTTCTGA | CATATTCCAGTCAAACCAAACG | 240, 500 | 0,364 | 0,48 | Relative plant height | A08 | [69] |
| HAU0091 | CTTCAAGGAGTCAGATTTGC | TTAAATCCTCACCGAGATGG | 310 | 0,48 | 0,364 | VW resistance | D04 | [60] |
| HAU1314 | GAAAAGCCCTTTACCAACAA | TCAGCTCTCCTATCTCACCTC | 150 | 0,375 | 0,500 | Fiber quality | A10 | [71] |
| HAU1332 | TTGGCATTGAGTACGCTTTA | TTGCTTCATTCGTAGTGCAT | 210 | 0,375 | 0,500 | Fiber quality | A04 | [72] |
| HAU1371 | GGGGTGTTTGGCTTATTAAA | AGAAGCGATATGAGGTCCAG | 305, 310, 315 | 0,314 | 0,391 | Fiber elongation | A06 | [73] |
| HAU2625 | CTGCCTTGCTCCTGCACCTT | GGGGTAAACAGGCGGGTGAG | 230, 240 | 0,364 | 0,480 | Fiber quality | D06 | [74] |
| HAU2768 | AGTGCCATCTGCTTCGGCTC | TGTGAACAATGAAAGTCTGACCCT | 210, 250, 275 | 0,314 | 0,391 | Fiber quality | A06 | [74] |
| JESPR095 | GCTTTTCTCGTAGACGTATG | GCATATTTATATACCAAGTCCCTC | 100 | 0,375 | 0,500 | - | A09 | [75] |
| JESPR114 | GATTTAAGGTCTTTGATCCG | CAAGGGTTAGTAGGTGTGTATAC | 90, 95 | 0,331 | 0,420 | Fiber quality | D09 | [76] |
| JESPR152 | GATGCACCAGATCCTTTTATTAG | GGTACTCGGAATCACAGTG | 200, 210, 230, 240 | 0,268 | 0,320 | salt_RPH, RSDM | D01 | [69,77] |
| JESPR204 | CTCCAGGTTCAATGGTCTG | GCCATGTTGGACAAGTAGTC | 150, 180, 205 | 0,293 | 0,357 | Seedling period, bud period, flower and boll period, salt tolerance, RSR (root-shoot ratio) | D13 | [63,78] |
| NAU0458 | AGGACTTGTCCACGTGCTTC | TTTGATTCTTTTCGGCTGCT | 180, 195 | 0,351 | 0,455 | Salt tolerance | D01 | [52,80] |

| | | | | | | | | | |
|---|---|---|---|---|---|---|---|---|---|
| NAU0837 | AACCAGCCAAATTTCATCAC | GATCCACGCCAACAAGTAA | 185, 210 | 0,331 | 0,420 | VW resistance | A06 | [60] |
| NAU0868 | GGCAAAACCATAAGGGTAAC | TAGCGTGAGATTGTGGCTTA | 190, 210 | 0,268 | 0,320 | Fiber quality | D03 | [76] |
| NAU0923 | GGAATTCAAGGTTGAAGGAG | GGAATTCAAGGTTGAAGGAG | 210, 230 | 0,372 | 0,495 | Fiber quality | D09 | [81] |
| NAU0934 | TGCTTTCGTATCCTTTTTCC | ATTAGAGAAGCCAGGGAGGT | 200 | 0,375 | 0,5 | salt_RMDA | A05 | [69] |
| NAU0990 | ACAGCGGTTCTTCTTTGTTC | TCGAAAACCTTCGGTGTAAG | 200, 220 | 0,351 | 0,455 | fiber strength | A01 | [62] |
| NAU1028 | CCGCCTAAGACTAATTGGAA | CAAATTGTAAGTGGCTGAGA | 210 | 0,375 | 0,500 | Plant height | A01 | [62] |
| NAU1042 | CATGCAAATCCATGCTAGAG | GGTTTCTTTGGTGGTGAAAC | 190, 210, 220, 230 | 0,342 | 0,438 | salt_RRDM, Total No. of sympodes | A05 | [69,77] |
| NAU1151 | TGGTTGCTTTGTATTGCTTG | CGTACTTGCGAAAGAGAACA | 145, 200 | 0,364 | 0,480 | Relative malondialdehyde | A12 | [69] |
| NAU1190 | CCATGTCCGTATCCATGTTA | TAAGGCAAGATAGGGTCAGG | 210, 220, 225 | 0,356 | 0,464 | Fiber quality | A03 | [81] |
| NAU1211 | CCTTCATTTCTCTCCTCCAA | GATACGAGGTCGTTTTGGTC | 235, 250, 850 | 0,370 | 0,491 | Fiber length (FL) | A12 | [62] |
| NAU1218 | TGTGATGAAGAACCCTCTCA | CACTCAACCCAATGAAACAA | 140 | 0,375 | 0,500 | Fiber quality | A06 | - |
| NAU1221 | CATGCAAATCCATGCTAGAG | AGGTTTCTTTGGTGGTGAAA | 210 | 0,375 | 0,500 | FOV resistance | D05 | [82] |
| NAU1230 | CATGCAAATCCATGCTAGAG | TCAAAAGGTTCTTTGGTGGT | 230, 250 | 0,375 | 0,500 | Fiber quality | A05 | [47] |
| NAU1255 | CATGCAAATCCATGCTAGAG | GGTTTCTTTGGTGGTGAAAC | 230, 240 | 0,375 | 0,500 | Fiber quality | D05 | [69] |
| NAU1269 | TACCTGAAACCCAAAATGGT | ACGCTGTTATAGGGCTCATC | 120, 150, 160 | 0,356 | 0,464 | Plant height | D05 | [72] |
| NAU2173 | GCCAAATAGGTCACACACAA | AGCGAGAAGGAGACAGAAAA | 210, 230 | 0,364 | 0,480 | Fiber quality, salt_RCC | D2 | [69] |
| NAU2265 | CAATCACATTGATGCCAACT | CGGTTAAGCTTCCAGACATT | 135, 145 | 0,375 | 0,500 | Fiber quality | A02/D03 | [79] |
| NAU2276 | CACATAATCGCCAACTTTGA | TCAAAACCCAACTCTTCTCC | 150 | 0,375 | 0,500 | - | - | [80] |
| NAU2317 | GACTCCAGCCTTCACACAT | TGGAAGAGTATAACGGCAGA | 225 | 0,375 | 0,500 | Fiber quality | A10 | [56] |
| NAU2437 | CTTGGAAAAAAGGAAGAGCAG | TTAAAAGACCAAAGGCAAGG | 220 | 0,375 | 0,500 | salt_RGR, RRDM, RGP, RMDA | D01 | [69] |
| NAU2508 | TGGAGGAGGGTGTAACATCT | GGCATTCAAGGAGATGAGTT | 145, 155 | 0,331 | 0,420 | SCpL (seed cotton locule) | A10 | [83] |
| CGR 5571 | TGAACATGGAAGTCCCACAA | GAAACTCGTGTGTCGCTTGA | 150, 160 | 0,375 | 0,500 | fiber strength | A01 | [62] |
| CGR6078 | CATGCAAGAAAGCTGCTCAA | TAGGCATGTGTCTCCGTGTG | 125, 130, 145 | 0,331 | 0,420 | Boll weight, fiber length, fiber strength, fiber elongation, fiber uniformity | A01 | [84] |
| CGR6103 | CAAAGGATGGGACACAGGTAA | TGCATTAGATACCGAAATGAGC | 120 | 0,268 | 0,320 | Fiber length | A08/D24 | [85] |

| | | | | | | | | |
|---|---|---|---|---|---|---|---|---|
| MGHES18 | GCCATCAATTGGTGAAGCAT | ATGCCTCGGTGAGAAAATTG | 180, 200 | 0,372 | 0,495 | VW resistance | A06 | [60] |
| TMB0426 | CAATCAGAGTGGGATGATGG | TGGAGTACGCGTTCAATGTG | 180 | 0,375 | 0,500 | resistance to reniform nematode | A11 | [86] |
| TMB1268 | CAGGTACCATTGATGCCAAA | CTCGAAACCTAGTGCCCTGT | 155 | 0,375 | 0,500 | Fiber uniformity ratio | D03 | [55] |
| TMB1740 | CACTCCAAACTCATCGCTCA | TGATTCATGATTCCCCCATT | 200 | 0,372 | 0,495 | VW resistance | A06 | [60] |
| MUSS162 | TTGGTTGGTTAATTACGGGG | GGCTTGTATCTCCCAGCAAG | 210 | 0,18 | 0,163 | BW, LI | A03 | [62] |
| MUSS193 | GAAAATGAGCACTTCTCCGC | AATGCGAATTGATCCAACAG | 200 | 0,375 | 0,500 | FL (12.9 cM), FM (2.22 cM), FU (6.20 cM), FE (19.71 cM) | A05 | [57] |
