# Supplementary material for: Genetic Diversity and Subspecific Races of Upland Cotton (Gossypium hirsutum L.)
Source: Genes (Basel). 2024 Nov 28;15(12):1533. doi: 10.3390/genes15121533 (PMC11675639; doi:10.3390/genes15121533)
Supplement: Supplementary file 1 [file genes-15-01533-s001.zip › genes-3293947-Table S2.pdf]

Table S2. Chromosomal locations and amplicon sizes of identified primers associated with fiber quality traits

№	Marker name	Forward primer (5'-3')	Reverse primer (5'-3')	Start-End point	Amplicon size (bp)	Chromosome
1	Gh034	ccctgttatctaacttctgttactcctaac	cctttgtttagctcttctatacttgaattcc	92944204-92944332	128	A13
2	Gh056	tccattagacaaagttttctaaagttc	tgagacttccaaccagatacag	8218577-8218701	124	D07
3	Gh064	gagaagccaatcccatttaaga	gatagtgcatctctagagaaggaca	2401747-2401871	124	D12
4	Gh110	accatcccaaagaatcatcctc	actaaaaccaaggcaataaagtg	65625666-65625790	124	D10
5	Gh132	tcatggaacaccaaagttgga	acatgatagattattcagcaatgca	115243512-115243657	145	A11
				64140098-64140276	178	D11
6	Gh247	cttcttccgccacgtaagtcc	cagcctaaccaagaacccaatcg	75788672-75788795	123	A09
				45693812-45693955	143	D09
7	Gh277	tactaaaaccaaggcaataaagtga	caccaccttccatatatcttgctc	65625667-65625868	201	D10
8	Gh388	catcatcatcgtcgtcgccg	gcaatggaagctttcgtctctttc	2358471-2358616	145	D05
9	Gh433	taccacattggatgtttgcaaaccc	atagcaaactggaatcactccaagc	126273963-126274146	183	A06
10	Gh591	gatttgaaaactggaggcatctcc	tcggttaccaccaatttaaccagc	-	-	-
11	DPL0050	ttactgaatcctaggcgagattgt	ttctagcctccacctcgct	11151383-11151522	139	A11
				10225403-10225554	151	D11
12	DPL0131	acatacgggttgaaatgtactcct	atgaatgcagatcattacgcct	4740756-4740945	189	A11
				4323044-4323264	220	D11
				4349765-4349984	219	D11
13	CIR0139	aaacaaatggagagggt	acctgtggtctgcaat	15592253-15592436	183	A05
				14239477-14239638	161	D05
14	CIR0246	ttagggtttagttgaatgg	atgaacacacgcacg	1054121-1054287	166	D02
15	CIR0329	tatatgtgaatggcccc	gcggattctgctgct	-	-	-
16	BNL0226	ttattctcacagccggaacc	ttcaccctctcgcttctcat	99367013-99367244	231	A03
				61346344-61346571	227	D02
17	BNL1421	tgaagatttggaggcaattg	gaaatcaagcctcaattcgg	6447563-6447753	190	A13
18	BNL1666	tgtcagaaaagtttttccaagg	agatcatatttaaaagaaaaagaaaacc	48402011-48402150	139	D01
19	BNL1694	cgtttgttttcgtgtaacagg	tggtggattcacatccaaag	40367154-40367393	239	A07
				27936319-27936542	223	D07
20	BNL2634	aacaacattgaaagtcgggg	cccagctgcttattggttc	-	-	-

21	BNL3140	caccattgtggcaactgagt	ggaaaagggaaagccattgt	42482196-42482302	106	D09
22	BNL3424	tgtgccgtctcaaaatgaag	aagaccaatctgttgccagc	107810726-107810878	152	A02
				393023-393189	166	D03
23	BNL3436	aacatagcctaccattgccg	ttgtttgccaaatttgaagc	2032359-2032556	197	D06
24	BNL3452	tgtaactgagcagccgtacg	gccaaagcagagtgagatcc	3846501-3846691	190	D05
25	BNL3594	agggattttgattgttgtgc	tgaattcaaaacaaatgttagcc	65409168-65409362	194	D06
26	BNL3601	ttccgttgatggaaattgaa	acaagaatgcgtgtgtctgc	11572754-11572928	174	D04
27	BNL3792	ttcgagatcccctgttctga	catattccagtcaaaccaaacg	80101745-80101974	229	A08
28	HAU0091	cttcaaggagtcagatttgc	ttaaatcctcaccgagatgg	72321631-72321929	298	A06
				38178388-38178703	315	D06
29	HAU1314	gaaaagccctttaccaacaa	tcagctctcctatctcacctc	77626452-77626584	132	A10
				24009041-24009159	118	D10
30	HAU1332	ttggcattgagtacgcttta	ttgcttcattcgtagtgcat	32289465-32289696	231	A04
31	HAU1371	ggggtgtttggcttattaaa	agaagcgatatgaggtccag	15781182-15781518	336	A06
				11368988-11369327	339	D06
32	HAU2625	ctgccttgctcctgcacctt	ggggtaaacaggcgggtgag	45683828-45684060	232	D06
33	HAU2768	agtgccatctgcttcggctc	tgtgaacaatgaaagtctgaccct	-	-	-
34	JESPR095	gcttttctcgtagacgtatg	gcatatttatataccaagtccctc	53732357-53732475	118	D09
35	JESPR114	gatttaaggtctttgatccg	caagggttagtaggtgtgtatac	-	-	-
36	JESPR152	gatgcaccagatccttttattag	ggtactcggaatcacagtg	-	-	-
37	JESPR204	ctccaggttcaatggtctg	gccatgttggacaagtagtc	118825913-118826070	157	A06
				28668607-28668793	186	D05
				59266490-59266676	186	D13
38	NAU0458	aggacttgtccacgtgcttc	tttgattcttttcggctgct	-	-	-
39	NAU0837	aaccagccaaatttcatcac	gatccacgccaacaagtaa	-	-	-
40	NAU0868	ggcaaaaccataagggtaac	tagcgtgagattgtggctta	16295996-16296208	212	A11
41	NAU0923	cctcttctttggctctgaaa	ggaattcaaggttgaaggag	73241002-73241230	228	A09
42	NAU0934	tgctttcgtatcctttttcc	attagagaagccagggaggt	108879239-108879444	205	A05
				2570699-2570914	212	D04
43	NAU0990	acagcggttcttctttgttc	tcgaaaaccttcggtgtaag	7316036-7316243	207	A02
				7305120-7305327	207	D02

44	NAU1028	ccgcctaagactaattggaa	caaattgtaagtggctgaga	103427290-103427532	242	A02
				4345089-4345319	230	D03
45	NAU1042	catgcaaatccatgctagag	ggtttctttggtggtgaaac	15809995-15810241	246	A05
				14406005-14406245	240	D05
46	NAU1151	tggttgctttgtattgcttg	cgtacttgcgaaagagaaca	-	-	-
47	NAU1190	ccatgtccgtatccatgtta	taaggcaagatagggtcagg	89890628-89890842	214	A03
48	NAU1211	ccttcatttctctcctccaa	gatacgaggtcgttttggtc	-	-	-
49	NAU1218	tgtgatgaagaaccctctca	cactcaacccaatgaaacaa	-	-	-
50	NAU1221	catgcaaatccatgctagag	aggtttctttggtggtgaaa	15809995-15810242	247	A05
				14406005-14406246	241	D05
51	NAU1230	catgcaaatccatgctagag	tcaaaaggttctttggtggt	-	-	-
52	NAU1255	catgcaaatccatgctagag	ggtttctttggtggtgaaac	15809995-15810241	246	A05
				14406005-14406245	240	D05
53	NAU1269	tacctgaaacccaaaatggt	acgctgttatagggctcatc	15810138-15810297	156	A05
54	NAU2173	gccaaataggtcacacacaa	agcgagaaggagacagaaaa	-	-	-
55	NAU2265	caatcacattgatgccaact	cggttaagcttccagacatt	107850525-107850760	235	A02
				345875-346094	219	D03
56	NAU2276	cacataatcgccaactttga	tcaaaacccaactcttctcc	12561268-12561501	233	A05
				11218700-11218933	233	D05
57	NAU2317	gactccagccttcacacat	tggaagagtataacggcaga	556970-557144	174	A10
				544473-544645	172	D10
58	NAU2437	cttggaaaaaaggaagagcag	ttaaaagaccaaaggcaagg	-	-	-
59	NAU2508	tggaggagggtgtaacatct	ggcattcaaggagatgagtt	117392994-117393150	156	A10
60	CGR 5571	tgaacatggaagtcccacaa	gaaactcgtgtgtcgcttga	-	-	-
61	CGR5597	acatggtgggaatgagaagc	aatgtagtgacgggcctttg	111153517-111153679	162	A01
62	CGR5602	atcgccattgtttactgcta	acccttccaccattaaaccc	15127641-15127799	158	D11
63	CGR6078	catgcaagaaagctgctcaa	taggcatgtgtctccgtgtg	115754546-115754665	119	A01
64	CGR6103	caaaggatgggacacaggtaa	tgcattagataccgaaatgagc	91189846-91189954	108	A08
65	MGHES18	gccatcaattggtgaagcat	atgcctcggtgagaaaattg	116462018-116462221	203	A06
				56470741-56470906	165	D06
66	TMB0426	caatcagagtgggatgatgg	tggagtacgcgttcaatgtg	75917022-75917226	204	A11

				45628409-45628607	198	D11
67	TMB1268	caggtaccattgatgccaaa	ctcgaaacctagtgccctgt	2072676-2072840	164	D03
68	TMB1740	cactccaaactcatcgctca	tgattcatgattcccccatt	127975269-127975425	156	A06
69	MUSS162	ttggttggttaattacgggg	ggcttgtatctcccagcaag	-	-	-
70	MUSS193	gaaaatgagcacttctccgc	aatgcgaattgatccaacag	4311376-4311579	203	A05
